# A V-to-F substitution in SK2 channels causes Ca^2+^ hypersensitivity and improves locomotion in a *C*. *elegans* ALS model

**DOI:** 10.1038/s41598-018-28783-2

**Published:** 2018-07-16

**Authors:** Young-Woo Nam, Saba N. Baskoylu, Dimitris Gazgalis, Razan Orfali, Meng Cui, Anne C. Hart, Miao Zhang

**Affiliations:** 1 Department of Biomedical and Pharmaceutical Sciences & Structural Biology Research Center, Chapman University School of Pharmacy, Irvine, California 92618 USA; 2 Department of Neuroscience, Brown University, Providence, Rhode Island 02912 USA; 3 Department of Pharmaceutical Sciences, Northeastern University School of Pharmacy, Boston, Massachusetts 02115 USA

## Abstract

Small-conductance Ca^2+^-activated K^+^ (SK) channels mediate medium afterhyperpolarization in the neurons and play a key role in the regulation of neuronal excitability. SK channels are potential drug targets for ataxia and Amyotrophic Lateral Sclerosis (ALS). SK channels are activated exclusively by the Ca^2+^-bound calmodulin. Previously, we identified an intrinsically disordered fragment that is essential for the mechanical coupling between Ca^2+^/calmodulin binding and channel opening. Here, we report that substitution of a valine to phenylalanine (V407F) in the intrinsically disordered fragment caused a ~6 fold increase in the Ca^2+^ sensitivity of SK2-a channels. This substitution resulted in a novel interaction between the ectopic phenylalanine and M411, which stabilized PIP_2_-interacting residue K405, and subsequently enhanced Ca^2+^ sensitivity. Also, equivalent valine to phenylalanine substitutions in SK1 or SK3 channels conferred Ca^2+^ hypersensitivity. An equivalent phenylalanine substitution in the *Caenorhabditis elegans* (*C*. *elegans*) SK2 ortholog *kcnl*-*2* partially rescued locomotion defects in an existing *C*. *elegans* ALS model, in which human SOD1G85R is expressed at high levels in neurons, confirming that this phenylalanine substitution impacts channel function *in vivo*. This work for the first time provides a critical reagent for future studies: an SK channel that is hypersensitive to Ca^2+^ with increased activity *in vivo*.

## Introduction

Calcium (Ca^2+^) mediates a variety of cellular signaling processes, including regulation of enzymatic activities, gene expression, synaptic transmission and ion channel activities^1,2^. Small conductance Ca^2+^-activated K^+^ (SK) channels are a unique group of ion channels that are activated exclusively by intracellular Ca^2+^ levels. The Ca^2+^-binding protein calmodulin (CaM) is constitutively associated with SK channels and Ca^2+^-binding by CaM activates these channels^3^. SK channels play a key role in the regulation of membrane excitability of neurons by Ca^2+^. In the central nervous system, activation of SK channels mediates the medium afterhyperpolarization (mAHP) and reduces the firing frequency of action potentials, thus contributing to regulation of neuronal excitability^4,5^.

In neurons, the Ca^2+^ sensitivity of SK channels is subject to negative modulation by neurotransmitters such as acetylcholine^6^ and norepinephrine^7^. Increased signaling by these neurotransmitters results in increased CaM phosphorylation at threonine 79 (T79). This phosphorylation can decrease the Ca^2+^ sensitivity of SK channels and, thus, increase neuronal excitability^3,8^. On the other hand, small molecules can increase the Ca^2+^ sensitivity of SK channels. For example, 1-ethyl-2-benzimidazolinone (1-EBIO)^9^ increases Ca^2+^ sensitivity of SK channels and reduces neuronal excitability^10^. And, NS309 (6,7-dichloro-1H-indole-2,3-dione 3-oxime) increases Ca^2+^ sensitivity of SK channels with a higher potency^11^.

ALS has commonalities with Spinal Muscular Atrophy (SMA), as both diseases result in spinal cord motor neuron degeneration and these diseases share other phenotypic, genetic, and molecular similarities. A missense mutation in the vesicle-associated membrane protein/synaptobrevin-associated membrane protein B (VAPB) gene causes both late-onset SMA and ALS^12^. These disorders may share a common neurodegenerative pathway and respond to similar treatments (e.g. riluzole). Previously, we reported that SK channels are genetic modifiers in vertebrate and invertebrate models of SMA^13^ and that SK channels are likely a critical target for the neuroprotective effects of riluzole in these models^14^.

Because of their critical roles in neuronal excitability, SK channels have been proposed as a drug target for motor neuron diseases and movement disorders^13–18^. Both SK2 and SK3 channel subtypes are expressed in the mammalian spinal motor neurons^19^. They play a critical role in the mAHP and the excitability of motor neurons^20–22^. Positive modulators of SK channels can also be used to regulate firing rates of cerebellar Purkinje cells. SK channel positive modulators Chlorzoxazone (CHZ), 1-EBIO and CyPPA normalize Purkinje cell firing and exert beneficial effects in mouse models of ataxia^16,17,23–25^. Riluzole showed promising results in recent phase II studies in a mixed population of ataxia patients^26^ and in inherited ataxia patients^27^; it was suggested that the ability of riluzole to facilitate SK channel activity was responsible for the beneficial impacts observed^28^.

**Figure 1:**
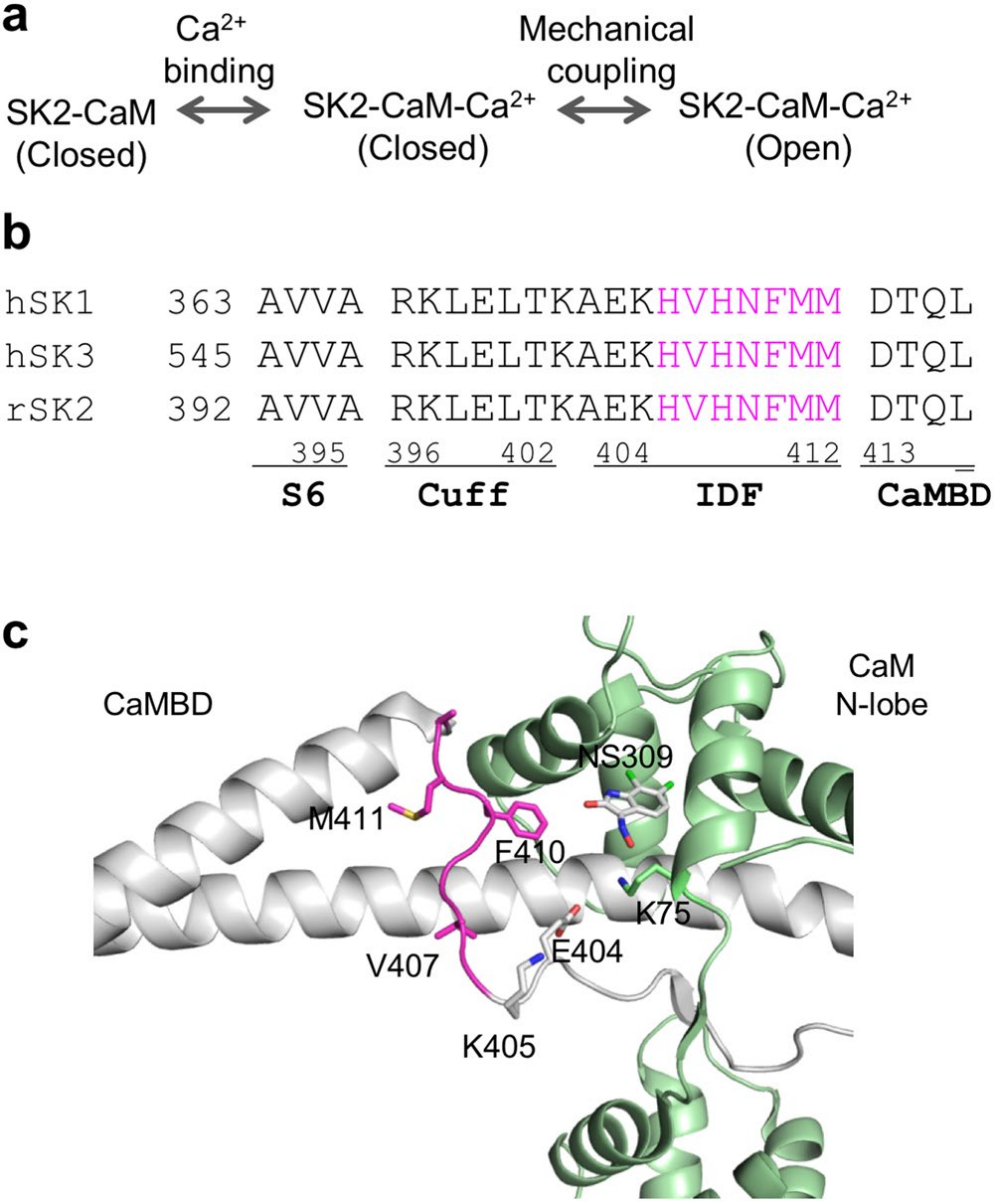
The SK channels IDF connects the CaMBD and transmembrane domain S6. (**a**) A simplified gating scheme for Ca^2+^-dependent SK channel activation may include two steps: (1) binding of Ca^2+^ to CaM associated with the SK channel and (2) mechanical coupling between Ca^2+^ binding to CaM and subsequent channel opening. (**b**) Amino acid sequence alignment of mammalian SK channels at the region connecting the CaMBD and the S6 transmembrane domain. Seven amino acid residues from the IDF shown in magenta are the focus of this study. Human and rat sequences are identical at this region. (**c**) Seven residues from the IDF (in magenta) are located between the CaMBD and the transmembrane S6 domain.

Yet, how SK channel Ca^2+^ sensitivity is modulated remains largely unclear, despite previous work^29–32^. Functional SK channels are tetrameric and composed of 4 channel subunits, like other voltage-dependent potassium channels. Each channel subunit contains six transmembrane α-helical domains that are denoted S1–S6. The Ca^2+^-sensor CaM associates with a channel CaM-binding domain (CaMBD), which is located within the channel C-terminus. Ca^2+^ binding to CaM induces conformational changes in both CaM and the channel CaMBD; these subsequently trigger opening of the channel pore^33–35^. A simplified gating scheme for Ca^2+^-dependent SK channel activation includes two steps: (1) binding of Ca^2+^ to CaM associated with the SK channel and (2) mechanical coupling between Ca^2+^ binding to CaM, CaMBD conformation change, and consequent channel opening (Fig. 1a). Thus, the Ca^2+^ sensitivity of SK channels could theoretically be modulated at either one of these two steps. Neither SK channel positive modulators (*e*.*g*. NS309) nor phosphorylation of CaM T79 influences the first step of the simplified gating scheme; *i*.*e*., Ca^2+^ binding to CaM-channel complex^30^. Instead, both SK channel positive modulators and phosphorylation of CaM T79 exert their modulation through the second step; *i*.*e*., mechanical coupling to channel opening. Previous mutagenesis and MD simulations work also established phosphatidylinositol 4,5-biphosphate (i.e. PIP_2_) as a critical factor in SK channel opening, modulation of SK channels by SK positive modulators^32^ and phosphorylation of CaM Thr79^31^.

We previously identified a flexible region (E404-M412) in the SK2-a channel that is essential for the second step of the hypothesized gating scheme and, thus, Ca^2+^ sensitivity of the SK2-a channel^30–32^. Flexible or intrinsically disordered fragments (IDFs) in proteins can be important for modulation of protein function^36–38^. We considered the possibility that the SK2-a IDF might play a critical role in regulation of SK channel Ca^2+^ sensitivity. Here, we report a valine to phenylalanine mutation in the IDF results in ~6 fold increase in SK2-a channel Ca^2+^ sensitivity. A combination of electrophysiological, X-ray crystallographic and computational approaches was utilized to determine the structural basis of SK2-a channel Ca^2+^ hypersensitivity. We found that the ectopic phenylalanine residue forms a novel interaction with methionine 411 in the IDF, stabilizes a putative PIP_2_-interacting residue lysine 405, and consequently increases channel Ca^2+^ sensitivity. We found that the equivalent mutation in the orthologous *Caenorhabditis elegans* (*C*. *elegans*) KCNL-2 protein partially rescues locomotion defects in a previously described model of superoxide dismutase (SOD1)-associated ALS. This result suggests that the valine to phenylalanine substitution increases Ca^2+^ sensitivity of SK channels *in vivo*. Combined, the studies reported here use biophysical, structural and genetic techniques, to develop a mutant SK channel that is hypersensitive to Ca^2+^.

## Results

### Mutagenesis of the residues in the IDF of SK2-a channel

In our previous study, we identified an IDF region (Fig. 1b, E404-M412) in the SK2-a channel that is essential for modulation of channel Ca^2+^ sensitivity^30–32^. IDF conformation is flexible and, thus, invisible in the apo-crystal structure, whereas the drug NS309 stabilizes IDF, resulting in a well-defined conformation in the crystal structure (Fig. 1c)^30^. One of the nine residues of the IDF, the negatively charged E404, forms a salt bridge with K75 of CaM. Mutation of E404 to other amino acids results in loss of SK2-a channel function^30^. K405 may be involved in the channel-PIP_2_ interactions and consequent modulation of SK2-a channel Ca^2+^ sensitivity^31,32^. Here, we investigate the remaining seven residues (Fig. 1b,c, H406-M412, highlighted in magenta) of the IDF, using site-directed mutagenesis, electrophysiological recordings, crystallography and molecular dynamic (MD) simulations.

**Figure 2:**
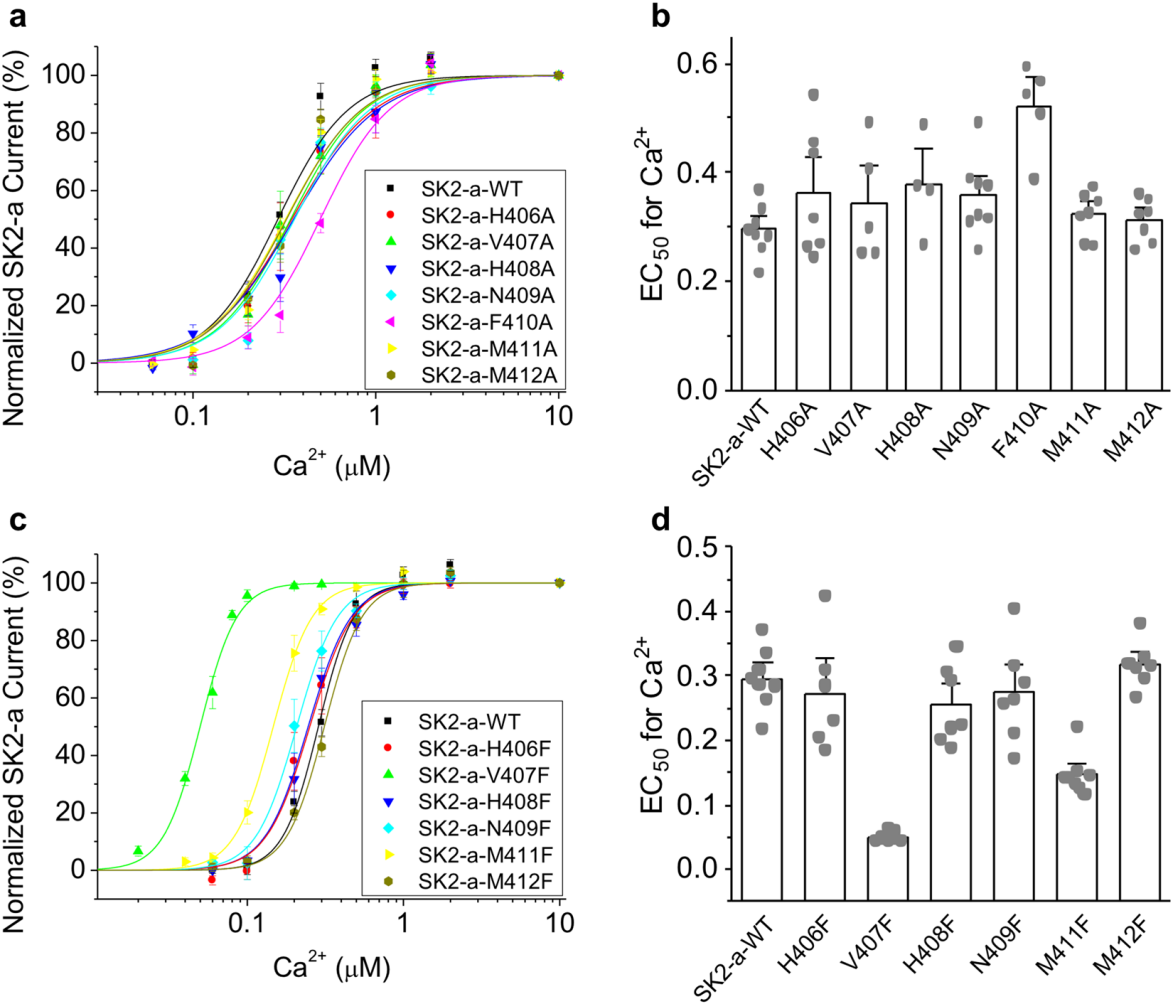
Mutations in the IDF change SK2-a channel Ca^2+^ sensitivity. (**a**) Dose-dependent activation by Ca^2+^ of the WT and alanine mutant SK2-a channels. (**b**) EC_50_ values for activation by Ca^2+^ of the WT and alanine mutant channels. (**c**) Dose-dependent activation by Ca^2+^ of the WT and phenylalanine mutant SK2-a channels. (**d**) EC_50_ values for activation by Ca^2+^ of the WT and phenylalanine mutant channels. Statistical analysis was performed using one-way ANOVA followed by Tukey’s post hoc tests. All data are presented as mean ± s.e.m.

We first determined if the side chains of these residues are important for the Ca^2+^ sensitivity of SK2-a channels, using an alanine scanning strategy (Fig. 2a). The effect of substituting alanine at each of the IDF seven residues on the Ca^2+^-dependent channel activation was examined using inside-out patch clamp recordings in into TsA201 cells. Compared with wild type (WT) SK2-a channel (EC_50_ = 0.30 ± 0.016 μM, n = 8), the alanine substitution at H406, V407, H408, N409, M411 or M412 did not change Ca^2+^-dependent activation of SK2-a currents. EC_50_ values for Ca^2+^ were 0.36 ± 0.058 μM (n = 7, P = 0.67), 0.34 ± 0.037 μM (n = 5, P = 0.95), 0.38 ± 0.045 μM (n = 4, P = 0.65), 0.36 ± 0.025 μM (n = 8, P = 0.73), 0.32 ± 0.021 μM (n = 7, P = 0.99) and 0.31 ± 0.020 μM (n = 7, P = 0.99), respectively. However, replacing aromatic amino acid F410 with an alanine (F410A) effectively reduced the SK2-a channel Ca^2+^ sensitivity (Fig. 2b). The F410A mutant channel was significantly less responsive to Ca^2+^, with an EC_50_ of 0.52 ± 0.036 μM (n = 5, P < 0.0001).

### The identification of a mutant SK2-a channel hypersensitive to Ca^2+^

As mutation of the only phenylalanine residue (F410) in the IDF resulted in reduced Ca^2+^ sensitivity, we hypothesized that introduction of an additional aromatic residue into the IDF might also change SK2-a channel Ca^2+^ sensitivity. We undertook phenylalanine scanning of the six IDF residues excluding F410 (Fig. 2c) and tested the effect of the phenylalanine substitutions on Ca^2+^-dependent channel activation using inside-out patches. Phenylalanine substitution at H406, H408, N409 and M412 did not change the activation of the SK2-a channel by Ca^2+^, compared to the WT channel, with EC_50_ values of 0.27 ± 0.036 μM (n = 6, P = 0.95), 0.25 ± 0.023 μM (n = 7, P = 0.55), 0.27 ± 0.037 μM (n = 7, P = 0.92) and 0.32 ± 0.013 μM (n = 7, P = 0.95), respectively. Changing M411 to phenylalanine modestly enhanced SK2–a channel Ca^2+^ sensitivity from 0.30 ± 0.016 μM (n = 8) to 0.15 ± 0.011 μM (n = 8, P < 0.0001). But, substituting a phenylalanine for hydrophobic V407 dramatically enhanced SK2-a channel Ca^2+^ sensitivity by almost six-fold, from 0.30 ± 0.016 μM (n = 8) to 0.051 ± 0.0024 μM (n = 8, P < 0.0001) (Fig. 2d).

We next examined the relationship between the size of the side chain at the residue 407 and SK2-a channel Ca^2+^ sensitivity. Amino acids of different sizes were introduced at position 407 by site-directed mutagenesis and their impact on SK2-a channel Ca^2+^ sensitivity was tested with inside-out patch recordings (Supplementary Fig. S1A). Neither alanine nor leucine substitution changed SK2-a channel Ca^2+^ sensitivity, with EC_50_ values of 0.34 ± 0.037 μM (n = 5, P = 0.49) and 0.30 ± 0.023 μM (n = 6, P = 0.97), respectively (Supplementary Fig. S1B). On the other hand, phenylalanine V407F or tryptophan V407W substitution strongly increased SK2-a channel Ca^2+^ sensitivity, with EC_50_ values of 0.051 ± 0.0024 μM (n = 8, P < 0.0001) and 0.059 ± 0.0062 μM (n = 6, P < 0.0001), respectively (Supplementary Fig. S1B). The impact of V407F or V407W was indistinguishable (P value of 0.99), suggesting that these substitutions at residue V407 were equally effective in enhancing SK2-a channel Ca^2+^ sensitivity.

### The structural insight into the Ca^2+^ hypersensitivity of the V407F mutant SK2-a channel

Next, we addressed how mutation of V407F might increase SK2-a channel Ca^2+^ sensitivity. First, we determined if the V407F substitution altered global conformation of the CaM–SK2 fragment complex using X-ray crystallography. CaM was co-crystallized with the mutant V407F SK2-a channel fragment, in the presence of Ca^2+^. The crystallographic data collection and refinement statistics are summarized in Supplementary Table S1.

**Figure 3:**
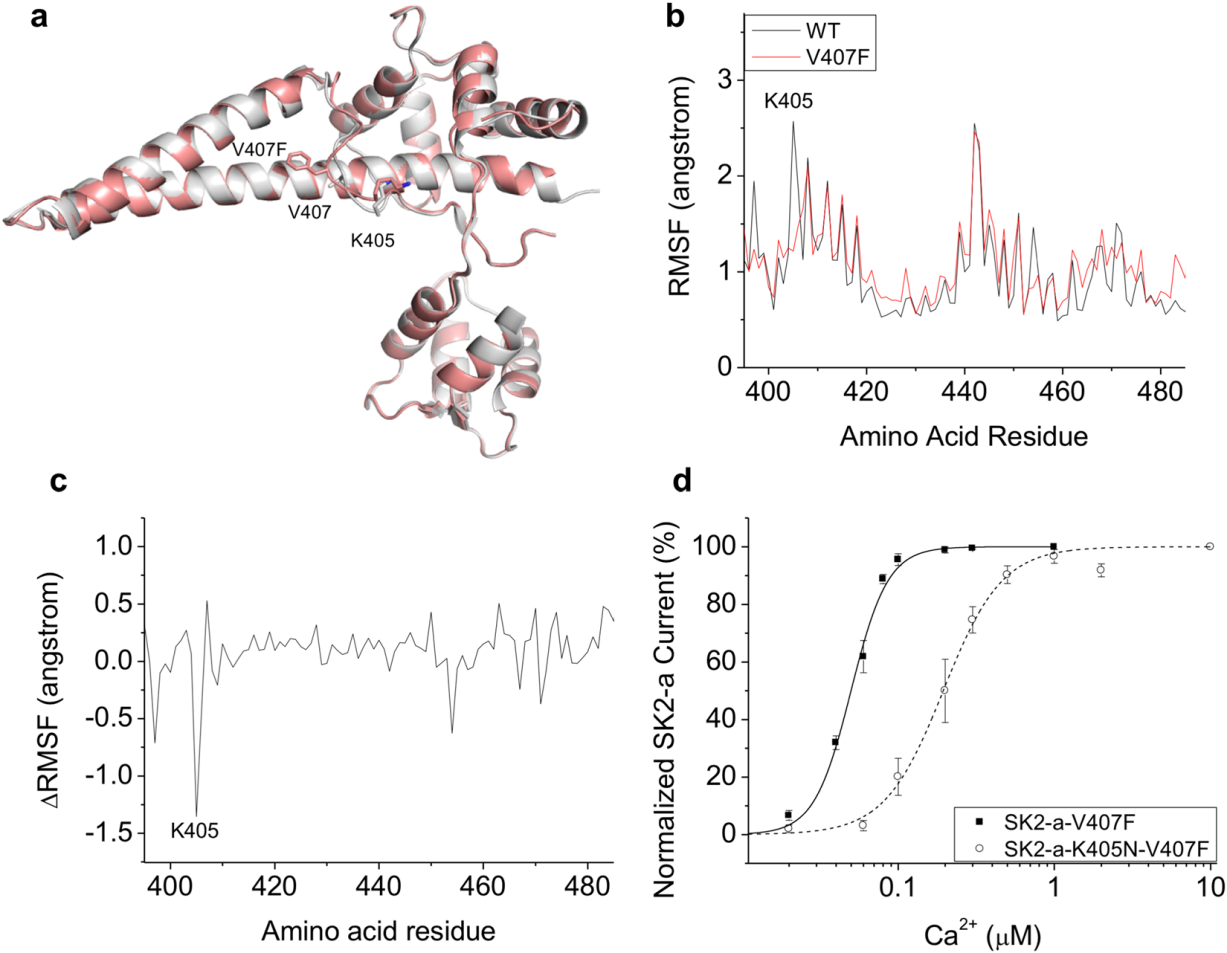
V407F stabilizes K405. (**a**) Overlaid crystal structures of WT (grey) and V407F (salmon) protein complexes show similar global conformation. (**b**) In MD simulations, the RMSF plot shows the difference in the structural flexibility of the residues between the WT and V407F mutant structures. (**c**) The difference RMSF plot (ΔRMSF = RMSF_(V407F)_ − RMSF_(WT)_) shows the reduced structural flexibility of K405 in the IDF of the V407F mutant structure. (**d**) Neutralizing mutation K405N right-shifts the dose-dependent activation of the V407F mutant SK2-a channel by Ca^2+^. Statistical analysis was performed using two-tailed *t*-test. All data are presented in mean ± s.e.m.

In the previously determined WT structure, the electron density for the IDF region was very poor (Supplementary Fig. S2A). The IDF was missing from the WT structure (PDB Code: 4J9Y). In the V407F mutant structure, the electron density for the IDF region was remarkably improved as a result of the stabilization (Supplementary Fig. S2B). Previously, we found that the IDF conformation in the WT structure can be stabilized by a small molecule called NS309 and determined through crystallography (PDB Code: 4J9Z). Similarly, in the V407F mutant structure, the electron density of the IDF was further improved by NS309 (Supplementary Fig. S2C). This V407F mutant structure with NS309 (PDB Code: 6ALE) was then compared with the WT structure with NS309 (PDB Code: 4J9Z). The V407F substitution did not dramatically shift global structure of complex, when compared the WT complex (Fig. 3a), with a root mean square deviation (RMSD) of 0.246 Å and 0.262 Å for CaM and the SK2 fragment, respectively. However, local differences exist between the two structures in the conformation of the IDF, with an RMSD of 0.798 Å.

We undertook MD simulations to compare the conformational dynamics of these two structures. The structural flexibility of the WT and V407F structures is shown as a plot of root mean square fluctuation (RMSF) results in Fig. 3b. Similar structural flexibility was observed in the rigid part (*e*.*g*. α-helices) of the protein complex between WT and V407F structures. On the other hand, the V407F mutant channel exhibited reduced fluctuations in the IDF region, with the largest difference at residue K405 (Supplementary Fig. S3A). The RMSF of K405 in the WT protein complex was 2.57 angstrom, whereas the RMSF of the same residue in the V407F mutant channel was 1.22 angstrom. The difference between the two RMSF data sets (ΔRMSF = RMSF_(V407F)_ − RMSF_(WT)_) is visible in Fig. 3c. The most prominent peak appears at the residue of K405 (Supplementary Fig. S3B). The negative peak amplitude of −1.35 angstrom indicates reduced structural flexibility (*i*.*e*. stabilization) of K405 in the mutant V407F complex, when compared with the WT complex.

Previously, we identified K405 as one of the positively charged residues that constitute a putative binding site for PIP_2_^31^. PIP_2_ is critical in mediating the mechanical coupling between Ca^2+^ binding to CaM and subsequent channel opening (Fig. 1a). MD simulations in the presence of PIP_2_ revealed the enhancement of PIP_2_ interaction energy in the V407F mutant structure compared to the WT structure (Supplementary Fig. S4). The median PIP_2_ interaction energies were −21.51 kcal/mol and −27.04 kcal/mol, in the WT and mutant structures, respectively. Hence, the Ca^2+^ hypersensitivity of the V407F mutant channel might be attributed to the enhanced mechanical coupling between Ca^2+^ binding and channel opening, as a result of K405 stabilization. We tested this hypothesis by introducing a neutralizing K405N mutation into the V407F mutant SK2-a channel. The double mutant (K405N/V407F) channel yielded a ~3.9 fold decrease in Ca^2+^ sensitivity (Fig. 3d), with an EC_50_ of 0.20 ± 0.023 μM (n = 7, P < 0.0001), compared to 0.051 ± 0.0024 μM (n = 8) for the V407F single mutant. In our previous report^31^, the K405N mutation in the WT SK2-a channel decreased Ca^2+^ sensitivity to 0.62 ± 0.046 μM (n = 6), which is a ~2 fold change from the WT. We conclude that the V407F mutant channel is more susceptible than the WT channel to the neutralizing impact of K405. The elevated Ca^2+^ sensitivity of the V407F mutant channel may be attributed, at least in part, to stabilizing K405 (Fig. 3b,c), a key residue for PIP_2_ interactions and subsequent mechanical coupling^31^.

In the V407F mutant structure, residues M411, V420, L463, K467 and Q470 are in the vicinity of the ectopic phenylalanine 407 (Supplementary Fig. S5A). We tested the effect of alanine substitutions of these five residues on the hypersensitivity of V407F mutant channel to Ca^2+^ activation using inside-out patches. Among these five residues tested, M411A mutation had the biggest impact. The double mutant channel V407F/M411A drastically decreased the Ca^2+^ sensitivity, with an EC_50_ of 0.21 ± 0.013 μM (n = 9, P < 0.0001), compared to 0.051 ± 0.0024 μM (n = 8) of the single V407F mutant (Supplementary Fig. S5B).

**Figure 4:**
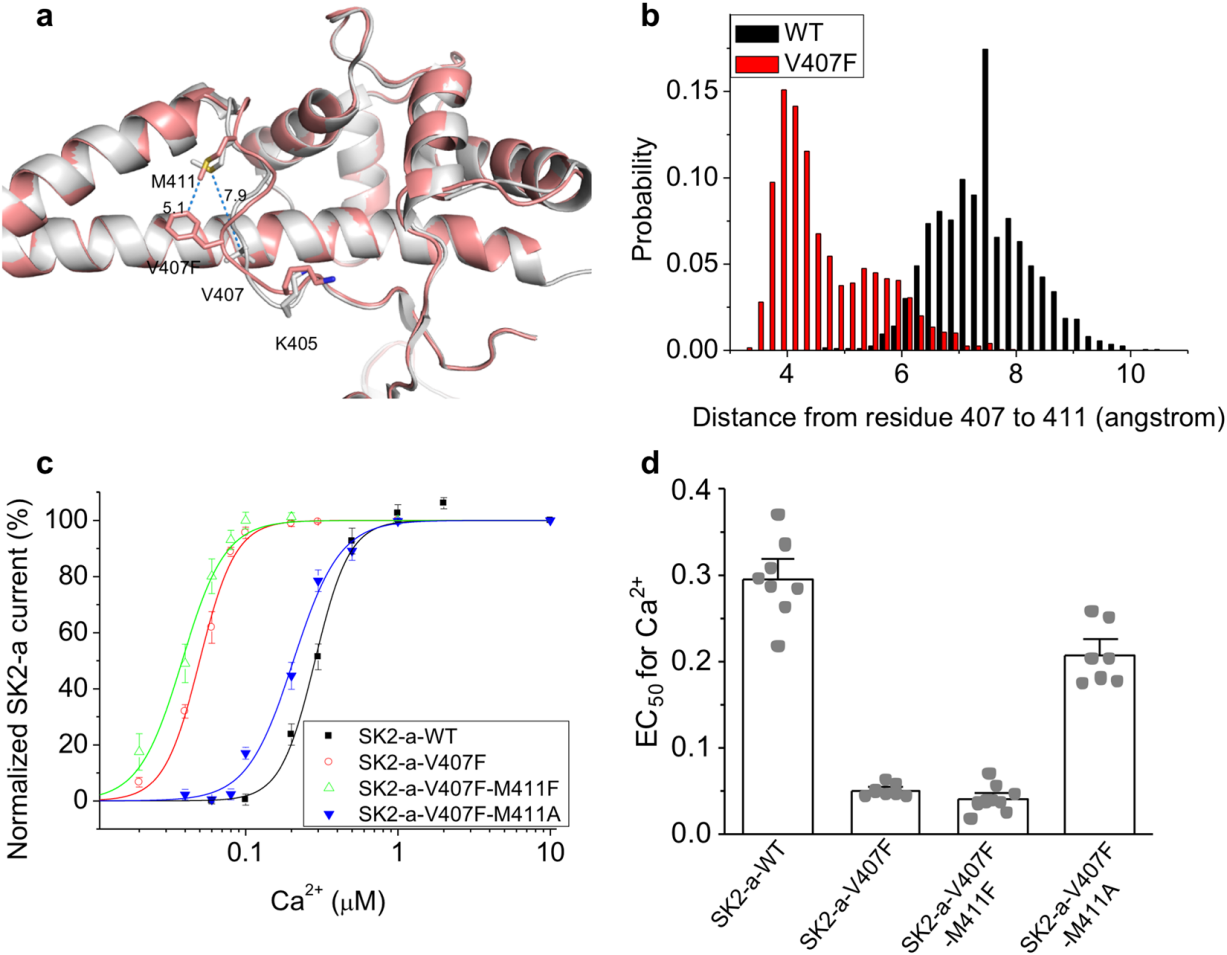
V407F interacts with M411. (**a**) Local conformational changes in the IDF of the V407F mutant crystal structure (salmon) compared to that of the WT structure (grey). (**b**) In MD simulations, the distance distribution histogram shows a shorter distance between residue 407 and residue 411 in the V407F mutant protein complex compared to the WT complex. (**c**) M411A mutation right-shifted the Ca^2+^ dependent activation of the V407F mutant SK2-a channel. (**d**) M411A mutation significantly compromised the Ca^2+^ hypersensitivity of the V407F mutant SK2-a channel. Statistical analysis was performed using one-way ANOVA followed by Tukey**’**s post hoc tests. All data are presented in mean ± s.e.m.

Next, we compared the IDF conformation in the WT and V407F mutant structures. When WT and V407F mutant protein complex structures are overlaid (Fig. 4a), V407 is 7.9 angstrom away from the IDF hydrophobic residue M411 in the WT structure, but the 407-to-411 distance is shortened to 4.7 angstrom in the V407F mutant structure. We used the MD simulations to explore changes in the dynamic interactions between amino acid residues, with particular focus on the IDF region. In the distance distribution histogram of MD simulations, the 407-to-411 median distance is 7.13 (V407-to-M411) angstroms for the WT structure (Fig. 4b). We note that M411A substitution did not change the Ca^2+^ sensitivity of the WT SK2-a channel (Fig. 2a,b), echoing the median 407-to-411 distance of 7.13 angstroms and the lack of prominent 407-to-411 interaction in the MD simulation of the WT structure (Fig. 4b). The distance distribution histogram also demonstrated a much shorter 407-to-411 median distance of 4.13 (F407-to-M411) angstroms for the mutant structure, indicating a strengthened hydrophobic interaction between the residues 407 and 411 (Fig. 4b). We calculated the interaction energies between the residues 407 and 411 using the Discovery Studio 2017 molecular modeling program (BIOVIA). The 407-to-411 interaction energies were −0.12 kcal/mol and −2.34 kcal/mol in the WT and mutant structures, respectively. We speculated that interruption of this 407-to-411 interaction through mutagenesis of M411 might compromise the Ca^2+^ hypersensitivity of the V407F mutant channel. To address this, we first introduced a phenylalanine at the location 411 in the V407F background, and resulted in the double mutant channel V407F/M411F. The double mutant did not exhibit significant change in the Ca^2+^ sensitivity (Fig. 4c), with an EC_50_ of 0.040 ± 0.0052 μM (n = 9, P = 0.89), compared to the single V407F mutant 0.051 ± 0.0024 μM (n = 8) (Fig. 4d). On the other hand, the double mutant channel V407F/M411A drastically decreased the Ca^2+^ sensitivity (Fig. 4d). Notably, a single M411A mutation did not change the Ca^2+^ sensitivity compared to the WT channel (Fig. 2b), suggesting that the side chain of M411 is required for interaction with the ectopic phenylalanine but not the original valine. Therefore, the V407F substitution results in formation of a new hydrophobic interaction with M411, which might drive stabilization of the IDF residues, especially PIP_2_-interacting K405 (Fig. 3c).

### The effectiveness of the equivalent valine to phenylalanine mutations in SK1 and SK3 channels

**Figure 5:**
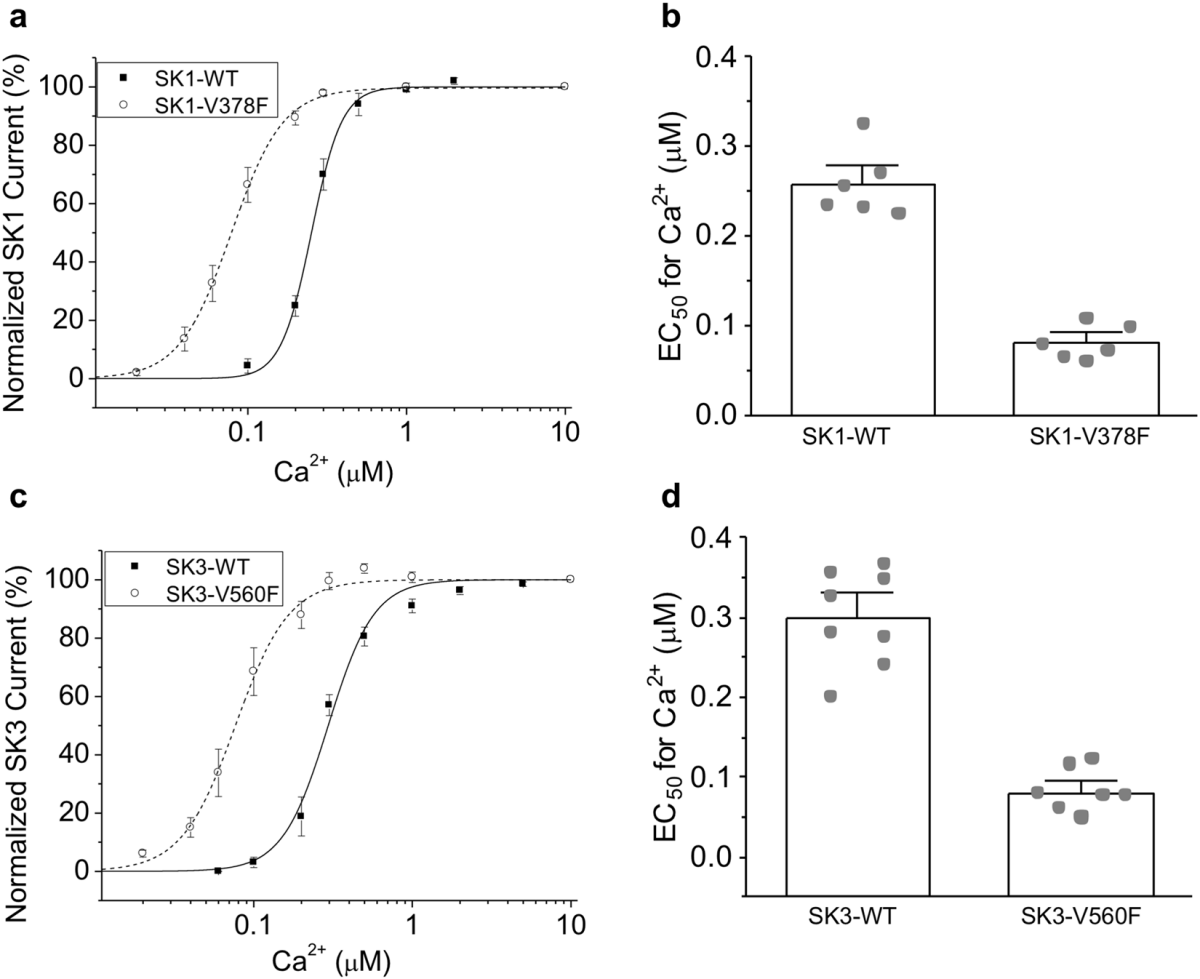
Phenylalanine substitution of the cognate valine in SK1 and SK3 channels increases Ca^2+^ sensitivity. (**a**) Dose-dependent activation of the WT and V378F mutant SK1 current by Ca^2+^. (**b**) EC_50_ values for the activation of the WT and mutant SK1 channels by Ca^2+^. (**c**) Dose-dependent activation of the WT and V506F mutant SK3 current by Ca^2+^. (**d**) EC_50_ values for the activation of the WT and mutant SK3 channels by Ca^2+^. Statistical analysis was performed using two-tailed *t*-test. All data are presented in mean ± s.e.m.

There are three mammalian SK channel subtypes expressed in the neurons (SK1, SK2, and SK3)^3^. IDF region amino acid sequences are completely identical in all three subtypes, including the valine corresponding to V407 (Fig. 1b). We determined if mutating these valines would change the Ca^2+^ sensitivity of SK1 and SK3 channels. Substitution of phenylalanine for V378 in the SK1 channel shifted the Ca^2+^ dose response curve to lower concentrations (Fig. 5a) and changed the EC_50_ value from 0.26 ± 0.017 μM (n = 6) to 0.081 ± 0.0079 μM (n = 6; P < 0.0001) (Fig. 5b). Substitution of phenylalanine for V560 in the SK3 channel has a similar effect (Fig. 5c). The EC_50_ values for Ca^2+^ activation of the WT SK3 and mutant SK3-V560F channels are 0.30 ± 0.024 μM (n = 8) and 0.080 ± 0.0095 μM (n = 8; P < 0.0001), respectively (Fig. 5d). Thus, substitution of phenylalanine at the cognate valine in all three SK channel subtypes effectively increases the Ca^2+^ sensitivity of these channels heterologously expressed in cell culture.

### Substitution of the cognate IDF valine in *C*. *elegans kcnl*-*2* ameliorates defects in a *C*. *elegans* model of SOD1 Amyotrophic Lateral Sclerosis

To address the impact of IDF substitutions and SK2 channel activation *in vivo*, we used a previously validated model of ALS, in which *C*. *elegans* expresses human superoxide dismutase (SOD1) in neurons^39^.

**Figure 6:**
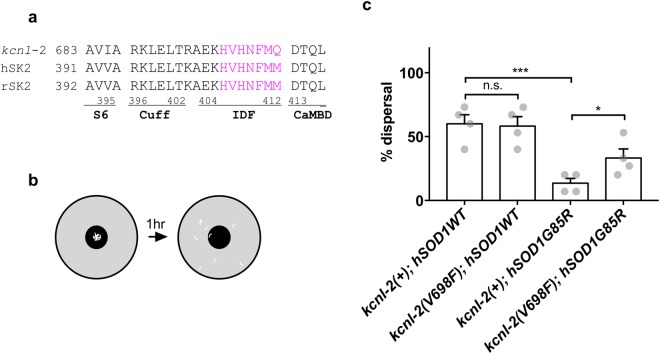
Phenylalanine substitution of the cognate valine in *C*. *elegans kcnl*-*2* ameliorates SOD1G85R ALS model locomotion defects. (**a**) Amino acid sequence alignment of mammalian SK2 channels and orthologous *C*. *elegans* KCNL-2 at the region connecting the CaMBD and the S6 transmembrane domain. Amino acids are numbered according to KCNL-2 isoform j. Seven amino acid residues from the IDF shown in magenta are the focus of this study. Human and rat SK2 sequences are identical in this region. (**b**) Diagram: to measure locomotion defects, adult animals were scored for dispersal 1 hour after they were placed at the center of a thin bacterial lawn on a 60-mm petri dish (food source). Coordinated animals disperse radially from the center of the bacterial lawn. Uncoordinated animals disperse more slowly. (**c**) Pan-neuronal expression of hSOD1G85R slowed dispersal, compared to hSOD1WT controls (one-tailed *t*-test, P = 0.0006; n = 60). Phenylalanine substitution of the cognate valine in *C*. *elegans kcnl*-*2* increased dispersal in hSOD1G85R animals, compared to hSOD1G85R animals carrying the *kcnl*-*2* wild type allele (one-tailed *t*-test, P = 0.0105; n = 60). Each determination is average of four trials. All data are presented in mean ± s.e.m. All animals were tested in *sod*-*1*(*tm776*) null background.

Animals expressing ALS mutant SOD1G85R-YFP fusion protein in all neurons (hSOD1G85R) have impaired locomotion, compared to animals expressing human wild-type SOD1-YFP (hSOD1WT) protein^39^. To determine the impact of SK2 channel activity on ALS-associated defects in this model, we re-created the V698F (equivalent to SK2-a V407F) mutation in the orthologous *C*. *elegans* gene, *kcnl*-*2* (Fig. 6a), using CRISPR/Cas9-mediated homologous recombination-based genome editing. To test the impact of *kcnl*-*2*(*V698F*) mutation on the locomotion of hSOD1WT or hSOD1G85R animals, we used a center-out dispersal locomotion assay (Fig. 6b). Most animals expressing hSOD1WT dispersed quickly, while animals expressing mutant hSOD1G85R were defective (n = 60; P = 0.0006) (Fig. 6c). The *kcnl*-*2*(*V698F*) mutation significantly increased dispersal in animals expressing hSOD1G85R, compared to hSOD1G85R animals carrying the wild type *kcnl*-*2* gene (n = 60; P = 0.0105) (Fig. 6c). Yet, *kcnl*-*2*(*V698F*) activity did not alter dispersal of hSOD1WT animals, consistent with a specific impact on animals expressing hSOD1G85R (Fig. 6c). Thus, *kcnl*-*2*(*V698F*) IDF mutation partially rescues locomotion defects in a *C*. *elegans* model of SOD1 ALS.

## Discussion

The SK channels are a unique group of potassium channels with an important role in regulating membrane excitability. These channels are activated exclusively by Ca^2+^-bound CaM^40^. Therefore, understanding the Ca^2+^ sensitivity of the CaM/SK complex is critical^8,31,35^. Previously described modifications usually decrease SK channel function. For example, phosphorylation of CaM threonine79 (T79) inhibits SK channels; this led to creation of the phosphomimetic CaM-T79D substitution that negatively modulates SK2-a channel Ca^2+^ sensitivity^8^. Or, in another example, an alternative RNA splice variants of rat or chicken SK2 channels (SK2-b) reduces channel Ca^2+^ sensitivity^35,41^. Also, disturbing positively charged residues in the putative SK2 PIP_2_ binding site decreases Ca^2+^ sensitivity^31,32^. But, to our knowledge there are no reports of SK channels with increased Ca^2+^ sensitivity. For the intermediate-conductance Ca^2+^-activated K^+^ (IK) channels, there are amino acid changes that can lock the channel into a low-conducting state with a high open probability, rather than the full conducting state^42^. Here, we report for the first time an alteration that increases SK channel sensitivity to Ca^2+^. The V407F substitution of SK2-a channel will facilitate future structural studies on the modulation of SK channel activity, as well as genetic and functional studies of SK channels and their impact in neurodegenerative diseases.

The V407F substitution is located in the SK2-a channel intrinsically disordered fragment (IDF), between E404 and M412 (Fig. 1b). Previous work demonstrated that IDF residue K405 is critical for the channel-PIP_2_ interactions^31^, when PIP_2_ mediates the mechanical coupling between Ca^2+^ binding to CaM and subsequent channel opening. The newly identified V407F substitution stabilizes K405 in MD simulations (Fig. 3c), reminiscent of the impact of the small molecule NS309 on SK channel IDF conformation^32^. In turn, stabilization of K405 enhances the mechanical coupling to channel opening (Fig. 1a), which is the likely mechanism for the Ca^2+^ hypersensitivity of the V407F mutant channels. Methionine 411 in the IDF seems to form a stronger hydrophobic interaction with the V407F mutation in the mutant channel structure, than with the V407 in the WT structure (Fig. 4a,b). The mutation of M411 to a phenylalanine also modestly increases the Ca^2+^ sensitivity of the SK2-a channel (Fig. 2c,d). The double mutant V407F/M411F, however, does not further enhance the Ca^2+^ sensitivity beyond the single V407F mutant (Fig. 4c,d), suggesting that one phenylalanine mutation at the residue 407 is sufficient to facilitate the 407-to-411 hydrophobic interaction. While the present study was in the final stage of review, cryo-electron microscopy (cryo-EM) structures of a human IK (also called SK4) channel have been reported^43^. Using the IK channel structure at the Ca^2+^-bound state (PDB code: 6CNN), we were able to generate a homology model of rat SK2 channel with the V407F substitution (Supplementary Fig. S6a). In this SK2 channel homology model, the N-lobe of CaM interacts with the S4–S5 linker of the SK2 channel in the presence of Ca^2+^. The CaMBD of the SK2 channel also forms substantial contacts with the S4–S5 linker in the presence of Ca^2+^. Interestingly, both residue 407 and residue 411 are located at the interface between CaMBD and the S4–S5 linker (Supplementary Fig. S6b). A top down view of the homology model shows the location of the two residues at the CaMBD-S4-S5 linker interface even more clearly (Supplementary Fig. S6c and d). The S4–S5 linker has been identified to be crucial for the Ca^2+^-dependent activation of the IK channel^43^. If the SK2 channel utilizes the same gating mechanism as IK channels, then the V407F substitution may enhance the hydrophobic interactions between the CaMBD and the S4–S5 linker, and thus increase the Ca^2+^ sensitivity of the SK2 channel. However, the possible interactions between the CaMBD and the S4–S5 linker and the effect of the valine to phenylalanine substitution on the interactions still need to be tested in the context of the SK2 channel. Equivalent valine to phenylalanine changes in SK1 and SK3 channels also confers Ca^2+^ hypersensitivity, addressing the conserved nature of IDF interactions (Fig. 5).

In resting neurons, the intracellular Ca^2+^ concentration is estimated to be ~0.1 μM^2^. As a result, the WT SK2-a channel (EC_50_ ~0.30 μM) is usually in the closed state in resting neurons, whereas the mutant V407F SK2-a channel (EC_50_ ~0.051 μM) could be in the activated state more than ~90% of the time, barring other regulatory events (Fig. 2c). To determine the *in vivo* consequences of SK2 channel activation in an intact nervous system, we turned to the model organism, *C*. *elegans*. The *C*. *elegans* genome contains four genes that encode SK family channels (*kcnl*-*1* through *kcnl*-*4*)^44^, but *kcnl*-*2* encodes the channel most similar to the mammalian SK2 channel. Previous work demonstrated that loss of *C*. *elegans kcnl*-*2* channel decreases sensitivity to compounds that directly modulate SK2 channel function (apamin and riluzole) and loss of *kcnl*-*2* function has behavioral consequences^14^. Loss of *kcnl*-*2* activity exacerbates neuromuscular defects in a *C*. *elegans* model of Spinal Muscular Atrophy and *kcnl*-*2* is required for the beneficial effects of riluzole in this model^14^. Riluzole increases SK2 channel activity, which may contribute to the modest benefits that riluzole provides to ALS patients.

Here, we used a *C*. *elegans* model of SOD1 ALS to examine the consequences of the cognate V698F mutation of *kcnl*-*2* in the intact nervous system. In this *C*. *elegans* ALS model, human SOD1 containing the patient G85R mutation is expressed at relatively high levels in *C*. *elegans* neurons (hSOD1G85R). In control animals, wild type human SOD1 protein is similarly expressed at high levels (hSOD1WT). Substitution of phenylalanine for the cognate valine in the *C*. *elegans* KCNL-2 IDF region had no obvious impact on *C*. *elegans* on locomotion, but did suppress locomotion defects in animals expressing SOD1G85R (Fig. 6c). These results are consistent with previous work on the role of SK2 channels in ALS and confirm that this phenylalanine substitution at this valine impacts channel function *in vivo*.

Given the importance of SK2 channels in neuronal excitability, understanding their regulation is critical. Here, we focus on the IDF fragment of SK2 and discover a phenylalanine to valine amino acid substitution that can increase SK2 channel Ca^2+^ sensitivity in tissue culture and also increases SK2 function *in vivo* in an animal model of ALS. This is the first SK channel variant with increased activity due to increased Ca^2+^ sensitivity that has been developed. Future studies can take advantage of these results to precisely dissect how SK channel activity contributes to neuronal activity under normal conditions and to neuron survival in neurodegenerative disease.

## Methods

### Electrophysiology

In our studies, the effect of mutations on the Ca^2+^ sensitivity of SK channels was investigated as previously described^29–31,45^. Briefly, mutations were introduced to the IDF region of rat SK2-a, human SK1 or human SK3 channels using QuickChange II site-directed mutagenesis kit (Agilent). The mutant channel cDNAs, along with CaM and GFP, at a ratio of 5:2.5:1 (weight), were transfected into TsA201 cells by the calcium–phosphate method. SK currents were recorded 1–2 days after transfection, with an Axon200B amplifier (Molecular Devices) at room temperature.

pClamp 10.5 (Molecular Devices) was used for data acquisition and analysis. The resistance of the patch electrodes ranged from 3–5 MΩ. The pipette solution contained (in mM): 140 KCl, 10 Hepes (pH 7.4), 1 MgSO_4_. The bath solution containing (in mM): 140 KCl, 10 Hepes (pH 7.2), 1 EGTA, 0.1 Dibromo-BAPTA, and 1 HEDTA was mixed with Ca^2+^ to obtain the desired free Ca^2+^ concentrations, calculated using the software by Chris Patton of Stanford University (http://www.stanford.edu/~cpatton/maxc.html). The Ca^2+^ concentrations were verified using Fluo-4 and standard Ca^2+^ buffers (Thermo Fisher Scientific).

Currents were recorded using an inside-out patch configuration. The intracellular face was initially exposed to a zero-Ca^2+^ bath solution, and subsequently to bath solutions with a series of Ca^2+^ concentrations. Currents were recorded by repetitive 1-s-voltage ramps from −100 mV to +100 mV from a holding potential of 0 mV. One minute after switching of bath solutions, ten sweeps with a 1-s interval were recorded. The integrity of the patch was examined by switching the bath solution back to the zero-Ca^2+^ buffer. Data from patches, which did not show significant changes in the seal resistance after solution changes, were used for further analysis. To construct the dose-dependent potentiation of channel activities, the current amplitudes at −90 mV in response to various concentrations of Ca^2+^ were normalized to that obtained at maximal concentration of Ca^2+^. The normalized currents were plotted as a function of the concentrations of Ca^2+^. EC_50_ values and Hill coefficients were determined by fitting the data points to a standard dose–response curve (*Y* = 100/(1 + (X/EC50) − Hill)). All data are presented in mean ± s.e.m. The data analysis was performed using pClamp 10.5 (Molecular Devices) in a blinded fashion. One-way ANOVA and Tukey’s post hoc tests were used for data comparison of three or more groups. The Student’s *t*-test was used for data comparison if there were only two groups.

### Protein crystallization and structure determination

The protein complex consisting of CaM and the SK2-a channel fragment (R395 - Q486 from rat SK2-a channel with a point mutation V407F) was purified as described in our previous papers^29–31,45^. Briefly, rat CaM was introduced into the pET28b(+) vector (Novagen) and expressed in Rosetta-2 *E*. *Coli*. cells (Novagen). The CaM protein was purified using a low substitution phenyl sepharose column (GE Healthcare). The mutant SK2-a channel fragment was also introduced into the pET28b(+) vector (Novagen) and the codons have been optimized to improve expression of this His-tagged protein in *E*. *Coli*. This His-tagged protein was expressed and purified using a Ni-NTA column. The purified mutant SK2-a channel fragment was mixed slowly with the purified CaM in the presence of Ca^2+^ to form a complex, followed by purification with a gel filtration column (GE Healthcare) pre-equilibrated in a solution with 10 mM Tris-HCl, 50 mM NaCl, and 10 mM CaCl_2_ (pH 7.5). The purified protein complex was concentrated to about 1 mM and then set up for crystallization. Protein crystals of the protein complex were grown in sitting drops by vapor diffusion at 20 °C. The complex (1 mM) was mixed in a 1:1 ratio with the reservoir solution, which consists of 1.4 M Li_2_SO_4_, 0.6 M (NH_4_)_2_SO_4_, 0.1 M sodium citrate, pH 5.6. Monoclinic crystals usually grew within 3 weeks. These preformed protein crystals were then incubated with SK channel modulator NS309 at its saturating concentrations for ~5 months, due to the poor water solubility of NS309. The soaked protein crystals were flash-cooled in liquid nitrogen for data collection, after a brief transfer to a suitable cryoprotectant (25% glycerol with the reservoir solution saturated with NS309). X-ray diffraction data were collected from single crystals on the X-ray diffraction system (D8 Venture Diffraction System, Bruker AXS Inc.) at our home institute and processed using PROTEUM2 (Bruker AXS Inc.). Initial phases were determined by molecular replacement (MR) using PHASER from the CCP4 suite. Our previously determined CaM/CaMBD complex structure (4J9Y) was used as a starting model to phase diffraction data. Solvent molecules were removed from the starting molecule before rigid body refinement. The crystallographic model was further constructed through iterative rounds of manual model-building using Coot and crystallographic refinement using REFMAC and PHENIX. The crystallographic statistics for data collection and model refinement are summarized in Supplementary Table S1. Structure graphics were created using PyMol (Schrödinger, LLC).

### Molecular Dynamics (MD) simulations

The structures of the WT or V407F protein complex were immersed in 0.15 M KCL solvation. The optimized potentials for liquid simulations (OPLS3) force field were used for the complexes, and the simple point charge (SPC) model was used for water molecules. All of the MD simulations were accomplished using the Desmond module of the Schrödinger software 2017–2 (Schrödinger, LLC). After being relaxed by Schrodinger Maestro’s default relaxation protocol which includes two stages of minimization (restrained and unrestrained) followed by four stages of MD runs with gradually diminishing restraints, the complexes were subjected to 100-ns MD simulations (NPT, constant Number, Pressure and Temperature ensemble) without any restraint at a constant temperature of 298 K. In total, 2000 snapshot structures were collected during the MD simulations. The root mean square deviation (RMSD), and the root mean square fluctuation (RMSF) of atomic positions in the trajectories were analyzed using the built-in utility of GROMACS program. RMSF calculations were performed using protein heavy atoms (including side chains) of the associated residues. The minimum distances between atoms of residues 407 and 411 in the trajectories were also analyzed using the built-in utility of GROMACS program.

The MD simulations in the presence of PIP_2_ were performed as previously described^31,32^. The interaction energies with PIP_2_ were acquired from MM-GBSA calculations using the thermal_mmgbsa.py script. The latter half of the trajectory was used in the MMGBSA calculations. Of the last thousand snap shots, every 10th snap shot was used as input for the input complexes. The VSGB solvent model was used for solvation model. OPLS3 was used to parameterize the protein and the ligand.

The Discovery Studio 2017 molecular modeling program (BIOVIA) was used to conduct energy minimization and calculate the interaction energy between the residues 407 and 411 from the WT and mutant crystal structures. The structures were subjected to energy minimization using Smart Minimizer algorithm (200 steps), and Generalized Born (GB) implicit solvent model using the CHARMM force field. Interaction energies between the residue pairs were calculated using a distance dependent dielectric constant (ε = 2r) implicit solvent model.

### Homology modeling

The full-length sequence of rat KCNN2 was retrieved from Universal Protein Resource ID Q9H2S1. The protein data bank structure ID 6CNN, a crystal structure of the human KCNN4 in complex with calmodulin, was selected as the template to build KCNN2^43^. Template biological assemblies were imported as heteromers into Maestro included within the Schrodinger 2018–1 software package. Structures were prepared for homology modeling using the protein preparation wizard included within the modeling suite. This corrected the model for missing loops, side chains, and bond orders. This tool was used to assign pronation states to amino acid residues and optimize hydrogen bond placement.

Homology modeling was performed using Prime program included in the Schrodinger 2018–1 software package. Sequence alignment to the template assemble was done using the ClustalW alignment method. Four calmodulin molecules were included in the KCNN2 model. Homology building was done in a series of steps where the backbone and side chain atom coordinates of conserved residues were copied over to the new model, followed by optimization of side chains, minimization of non-template residues, and building insertions and deletions in the alignment. Non-template loops are refined using the ultra-extended loop sampling method. The model is then checked for missing atoms and minimized.

### Construction of guide RNA plasmid

Embryonic U6 promoter-driven *kcnl*-*2* guide RNA was amplified from the *pU6*::*klp*-*12*::*sgRNA*^46^ vector template using *kcnl*-*2* targeting primers: *kcnl*-*2_guide_f* and *kcnl*-*2_guide_r*. The resulting linear DNA was circularized to generate the final construct pHA0815 *pU6*::*kcnl*-*2::guide*.

### Strain construction

*kcnl*-*1* V698F point mutation, the cognate of SK2-a V407F, was introduced into the endogenous *kcnl*-*2* gene using CRISPR/Cas9-mediated homologous recombination (HR) with *pha*-*1* co-conversion^47^. In brief, co-editing of a temperature-sensitive, lethal mutation at the unlinked *pha*-*1* gene facilitates identification of animals with CRISPR/Cas9-mediated genomic editing events in *kcnl*-*2*. The guide RNA construct pHA0815 *pU6::kcnl*-*2::guide* was injected at 50 ng/μl into temperature-sensitive *pha*-*1*(*e2123*)*III* animals raised at 15 degrees with 50 ng/μl of *Peft*-*3::Cas9* plasmid^46^, 50 ng/μl of *pha*-*1 sgRNA* + *Cas9* pJW1285 plasmid^47^,10 μM of 200mer sense *pha*-*1*(+) rescue oligonucleotide^47^ and 10 μM of the single-stranded oligonucleotide template (ssODN) *kcnl*-*2_ssODN*. The *kcnl*-*2_ssODN* contained synonymous mutations to prevent further genomic editing and to generate an ApaLI restriction site for PCR genotyping. Injected animals were transferred to a restrictive temperature at 25 degrees; all surviving progeny were screened for potential HR events by PCR genotyping with primers *kcnl*-*2_scr_f* and *kcnl*-*2_scr_r*, followed by ApaLI restriction digestion. Editing events were verified by sequencing and the strain was backcrossed four times to wild type N2 before analysis. The *pha*-*1* locus was sequenced in backcrossed lines to verify the presence of the wild-type *pha*-*1*(+) allele. The *kcnl*-*2*(*rt462[V698F]*) allele was crossed into animals expressing human wild-type SOD1-YFP (hSOD1WT) or human ALS-associated SOD1G85R-YFP (hSOD1G85R) under the pan-neuronal *snb*-*1* promoter^39^ in *sod*-*1*(*tm776*) background.

### Dispersal Assay

Dispersal was scored on 60-mm diameter petri dishes freshly spread with 200 μl of thin OP50 bacterial lawn (food source) on 10 ml of Nematode Growth Medium (NGM). Day 1 adult animals were placed at the center of the bacterial lawn and percentage of animals escaping the 15-mm diameter central ring is reported. One-way ANOVA and Sidak’s tests were used for data comparison. All experiments were carried out in accordance with relevant guidelines and regulations. When appropriate, protocols were approved by institutional committees.

### Primers

*kcnl-2_guide_f: GAATTGACAAGTTTTAGAGCTAGAAATAGC, kcnl-2_guide_r: CAACTTTCTCAAACATTTAGATTTGCAATTC, kcnl-2_ssODN: CTACCTGCTTAGTAAGTTGAGTGTCTTGCATGAAATTGTGGAAATGCTTCTCTGCGCGTGTCAATTCCAACTTTCTCGCAATAACCGCGACAACCATCGAAG, kcnl-2_scr_f: CCATTTGGCTTGTTGCTATC, kcnl-2_scr_r: AATTACCTTTGCCACGTCTC.*

### Strains

HA2614: *kcnl-2(+)I; sod-1(tm776)II; pha-1(+)III; [hSOD1WT::YFP]*

HA3351: *kcnl*-*2*(*rt462[V698F]*)*I; sod*-*1*(*tm776*)*II; pha*-*1*(+)*III; [hSOD1WT::YFP]*

*HA2612: kcnl*-*2*(+)*I; sod*-*1*(*tm776*)*II; pha*-*1*(+)*III; [hSOD1G85R::YFP]*

HA3397: *kcnl*-*2*(*rt462[V698F]*)*I; sod*-*1*(*tm776*)*II; pha*-*1*(+)*III; [hSOD1G85R::YFP].*

### Experimental Design and Statistical Analysis

One-way ANOVA and post hoc tests were used for data comparison of three or more groups. The Student’s *t*-test was used for data comparison if there were only two groups.

### Data availability

The structure coordinates have been deposited in the Protein Data Bank under accession codes 6ALE and 6CZQ.

## Electronic supplementary material

Supplementary Information
