# Supplementary material for: A V-to-F substitution in SK2 channels causes Ca2+ hypersensitivity and improves locomotion in a C. elegans ALS model
Source: Sci Rep. 2018 Jul 16;8:10749. doi: 10.1038/s41598-018-28783-2 (PMC6048120; doi:10.1038/s41598-018-28783-2)
Supplement: Supplementary file 1 — Supplementary Information [file 41598_2018_28783_MOESM1_ESM.pdf]

Supplementary Information

A V-to-F substitution in SK2 channels causes Ca^{2+} hypersensitivity and improves locomotion in a *C. elegans* ALS model

Young-Woo Nam [1], Saba N. Baskoylu [2], Dimitris Gazgalis [3], Razan Orfali [1], Meng Cui [3], Anne C. Hart [2], Miao Zhang [1,*]

[1] Department of Biomedical and Pharmaceutical Sciences & Structural Biology Research Center, Chapman University School of Pharmacy, Irvine, California 92618, USA

[2] Department of Neuroscience, Brown University, Providence, Rhode Island 02912, USA

[3] Department of Pharmaceutical Sciences, Northeastern University School of Pharmacy, Boston, Massachusetts, 02115, USA

[*] Correspondence should be addressed to M.Z. (zhang@chapman.edu)

Running title: A mutant SK2 channel hypersensitive to Ca^{2+}

Email: zhang@chapman.edu
Phone: 1-714-516-5478
Department of Biomedical and Pharmaceutical Sciences
Chapman University School of Pharmacy
9501 Jeronimo Road
Irvine, CA 92618

Table S1. Crystallographic statistics

**Data Collection**[a]		
	**V407F 6CZQ**	**V407F with NS309 6ALE**
Space Group Unit Cell Dimensions	C2 a=76.3 Å, b=66.7 Å, c=64.7 Å α=90.0°, β=92.2°, γ=90.0°	C2 a=76.6 Å, b=66.9 Å, c=64.7 Å α=90.0°, β=92.5°, γ=90.0°
Wavelength (Å)	1.08	1.08
Resolution range (Å)	26.9-2.20 (2.23-2.20)	29.72-2.50 (2.59-2.50)
Completeness (%)	99.8 (99.81)	100 (100)
Total Observations	111,152 (10,843)	123,879 (12,303)
Unique Observations	16,556 (1,621)	11,420 (1,128)
Mean Redundancy	6.7(6.7)	10.8 (10.9)
Mean I/σ(I)	14.3 (4.3)	13.3 (5.4)
R_{merge}[b]	0.089 (0.435)	0.141 (0.513)
R_{pim}[c]	0.037 (0.182)	0.045 (0.161)
**Model Refinement**[a]		
Resolution Range (Å)	25.1-2.2 (2.28-2.2)	23.85-2.5 (2.589-2.5)
No. reflections	16,534 (1,618)	11,410 (1,121)
R_{work}[d]	0.1977(0.232)	0.188 (0.249)
R_{free}[d]	0.249 (0.2796)	0.255 (0.289)
No. atoms / Avg. B (Å^2)	2,087/45.18	2,096/45.48
protein	1,938/42.9	1,938/44.6
calcium ions	2/59.8	2/59.8
solvent	116/40.2	116/40.2
ligand	17/84.5	42/100
Phi/Psi angles favored (%) / outliers (#)	99.58/ 0	99.15/ 0.84
r.m.s.d. bond angles (°)	0.008	0.007
r.m.s.d. bond lengths (Å)	0.86	1.13

[a] Values in parentheses refer to data in the highest resolution shell.

[b] $R_{merge} = \sum_{hkl}\sum_j |I_j - \langle I\rangle| / \sum_{hkl}\sum_j I_j$. $\langle I\rangle$ is the mean intensity of j observations of reflection *hkl* and its symmetry equivalents.

[c] R_{pim} (precision-indicating merge) = $\sum_{hkl} (1/n_{hkl} - 1)^{1/2} \sum_j |I_j - \langle I\rangle| / \sum_{hkl}\sum_j I_j$. n is the number of observations of reflection hkl.

[d] $R_{cryst} = \sum_{hkl} |F_{obs} - kF_{calc}| / \sum_{hkl} |F_{obs}|$. $R_{free} = R_{cryst}$ for 5% of reflections excluded from crystallographic refinement.

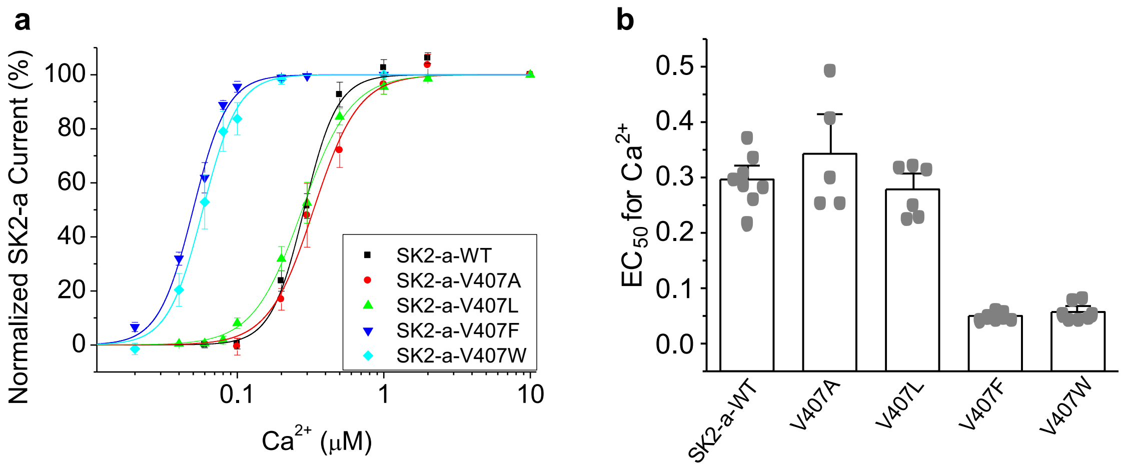

Fig. S1. Mutation of V407 to an aromatic residue increases SK2-a channel Ca^{2+} sensitivity. (a) Ca^{2+}-dependent activation of the WT and mutant SK2-a channels with mutations at residue V407. (b) EC_{50} values for the activation by Ca^{2+} of the WT and mutant channels. Statistical analysis was performed using one-way ANOVA followed by Tukey's post hoc tests. All data are presented as mean ± s.e.m.

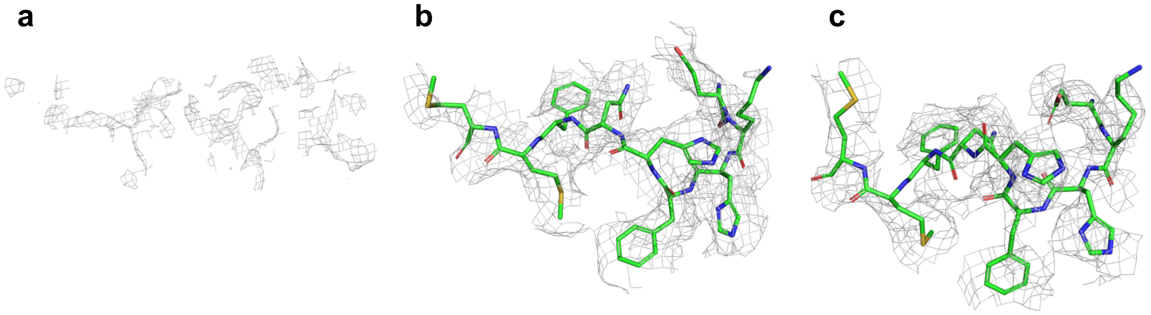

Fig. S2. Electron density map of the IDF region. (a) Electron density map (grey, 2|F_o|-|F_c|) of the IDF in the WT crystal structure (PDB Code: 4J9Y). (b) Electron density map (grey, 2|F_o|-|F_c|) of the IDF in the V407F mutant crystal structure (PDB Code: 6CZQ). (c) Electron density map (grey, 2|F_o|-|F_c|) of the IDF in the V407F mutant crystal structure (PDB Code: 6ALE) in the presence of NS309. The maps are contoured at 0.8 σ.

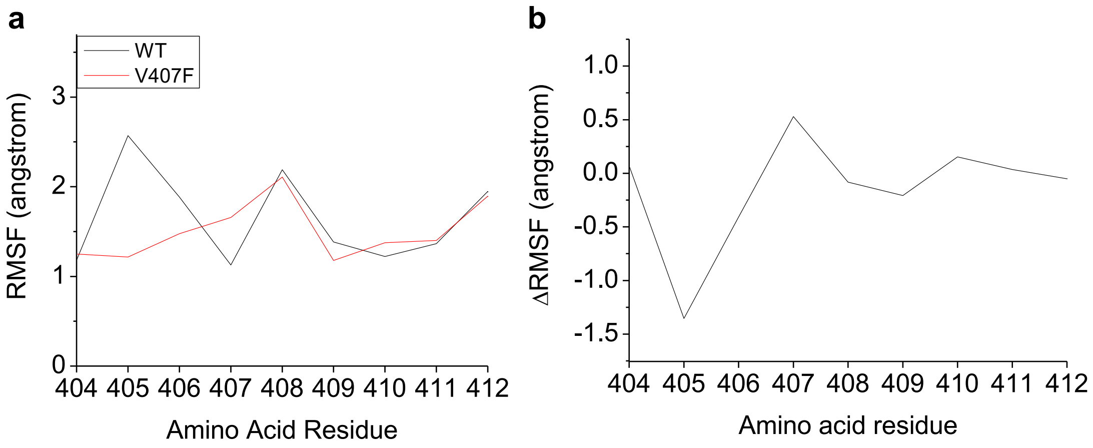

Fig. S3. The V407F mutation affects the flexibility of the IDF residues. (a) In MD simulations, the RMSF plot shows the difference in the structural flexibility of the IDF residues between the WT and V407F mutant structures. (b) The difference RMSF plot ($\Delta RMSF = RMSF_{(V407F)} - RMSF_{(WT)}$) shows changes of structural flexibility in the IDF.

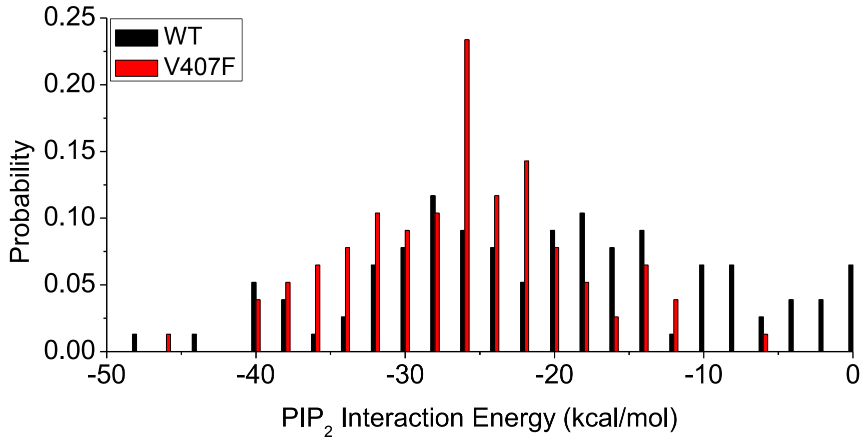

Fig. S4. The enhancement of PIP_2 interaction energy in the V407F mutant structure compared to the WT structure in MD simulations.

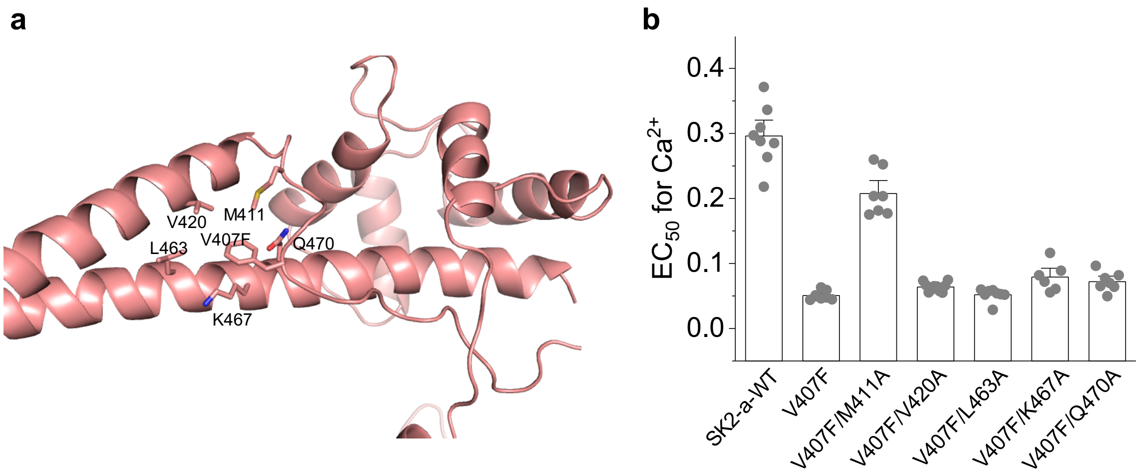

Fig. S5. The residues in the vicinity of the ectopic phenylalanine 407. (a) In the V407F crystal structure, the residues shown as sticks may potentially form hydrophobic interactions with the aromatic ring of the F407. (b) In electrophysiological recordings, the M411A mutation has the largest impact on the EC_{50} for Ca^{2+} of the V407F mutant channel.

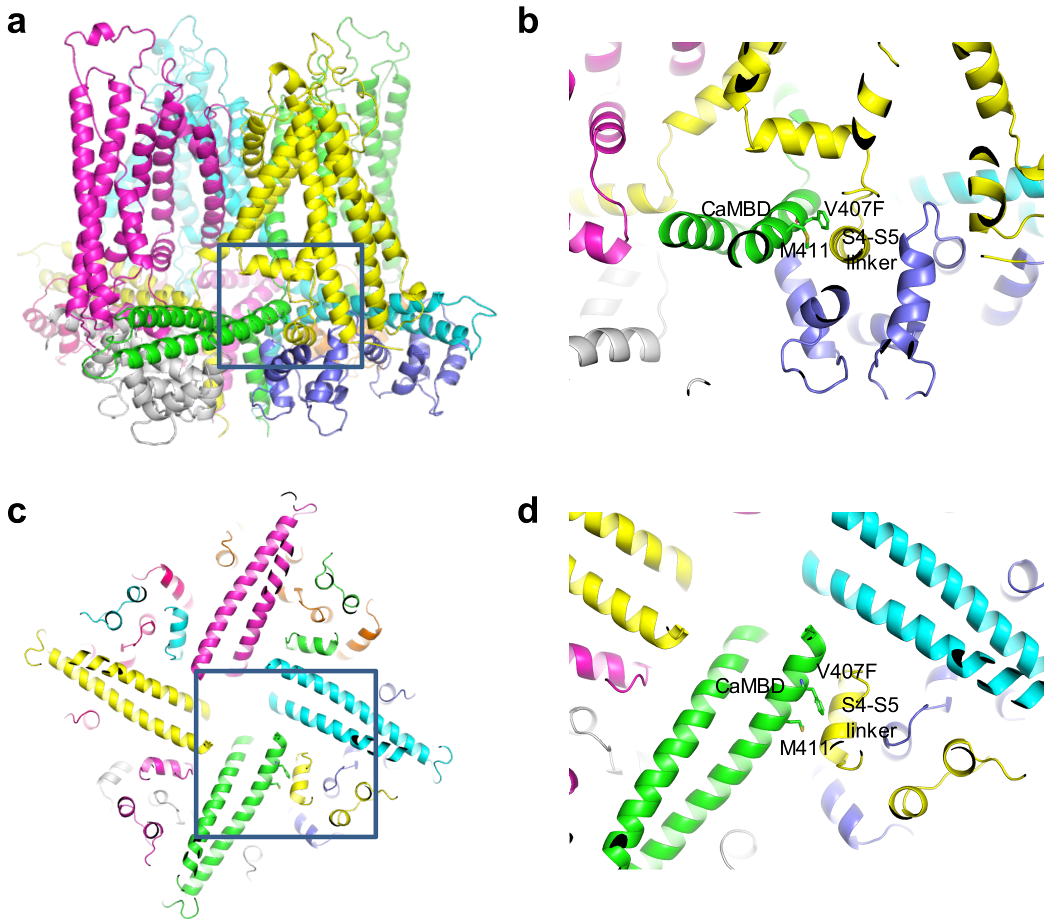

Fig. S6. The homology model of the mutant V407F SK2 channel. (a) The side view of the mutant V407F SK2 channel. (b) A close-up view of the interface between the CaMBD and the S4-S5 linker. (c) The top-down view of the intracellular portion of the mutant V407F SK2 channel. (d) The residues 407 and 411 are located at the interface between the CaMBD and the S4-S5 linker.
